# Development of the electric organ in embryos and larvae of the knifefish, *Brachyhypopomus gauderio*

**DOI:** 10.1016/j.ydbio.2020.06.010

**Published:** 2020-10-01

**Authors:** Ilham J.J. Alshami, Yosuke Ono, Ana Correia, Christian Hacker, Anke Lange, Steffen Scholpp, Masashi Kawasaki, Philip W. Ingham, Tetsuhiro Kudoh

**Affiliations:** a Biosciences, College of Life and Environmental Sciences, University of Exeter, Exeter, EX4 4QD, UK; b Living Systems Institute, University of Exeter, Exeter, EX4 4QD, UK; c Department of Physiology, Development and Neuroscience, University of Cambridge, CB2 3EG, UK; d Department of Biology, University of Virginia, Charlottesville, VA, 22904, USA; e Lee Kong Chian School of Medicine, Nanyang Technological University, Singapore

**Keywords:** Electric fish, Electric organ, Electrocyte, Staging, Knifefish

## Abstract

South American Gymnotiform knifefish possess electric organs that generate electric fields for electro-location and electro-communication. Electric organs in fish can be derived from either myogenic cells (myogenic electric organ/mEO) or neurogenic cells (neurogenic electric organ/nEO). To date, the embryonic development of EOs has remained obscure. Here we characterize the development of the mEO in the Gymnotiform bluntnose knifefish, *Brachyhypopomus gauderio.* We find that EO primordial cells arise during embryonic stages in the ventral edge of the tail myotome, translocate into the ventral fin and develop into syncytial electrocytes at early larval stages. We also describe a pair of thick nerve cords that flank the dorsal aorta, the location and characteristic morphology of which are reminiscent of the nEO in Apteronotid species, suggesting a common evolutionary origin of these tissues. Taken together, our findings reveal the embryonic origins of the mEO and provide a basis for elucidating the mechanisms of evolutionary diversification of electric charge generation by myogenic and neurogenic EOs.

## Highlights

• Developmental staging of the electric bluntnose knifefish, *Brachyhypopomus gauderio* embryos and larvae.
• The primordia of the myogenic electric organ originate in the ventral somite, migrate in the ventral fin and develop to the electric organ.
• Evolutionary conservation between the nerve codes in the B. gauderio and the neurogenic electric organs in the Apteronotidae.

## 1 Introduction

Electric fish can generate electric fields in the water allowing them to sense their world in darkness and connect with potential mates and competitors. They do so using electric organs (EOs) that are specialized in generating and discharging electricity. It is thought that during fish evolution, EOs have arisen in multiple lineages, such as the catfish, the South American electric fishes (Gymnotiformes), the African weakly electric fishes (Mormyroidae), and in two groups of cartilaginous fish, the electric rays and the skates (Moller and Bauer, 1973; Belbenoit et al., 1979; Kirschbaum and Schwassmann, 2008; Zakon et al., 2008; Macesic and Kajiura, 2009; Nelson, 2011).

Development of EOs from larval to adult stages has been studied in a variety of fish species (Denizot et al., 1978, 1982; Kirschbaum and Schwassmann, 2008; Schwassmann et al., 2014). EOs are classified as myogenic EOs (mEOs) or neurogenic EOs (nEOs) depending on their cell types. Kirschbaum and Schwassman (2008) described the larval and juvenile development of the EOs from eight species of gymnotiforme knifefish, and compared their morphology and location among the species. These gymnotiform knifefish develop mEOs on the ventral side of or within the myotome, depending on species. In addition, Apteronotid species also develop the nEO either side of the dorsal aorta. During development of the nEO from larval to adult stage of Apteronotids, the tissue undergoes massive expansion to form a mature nEO by the adult stage. At the same time, mEOs in these species diminish before the adult stage, suggesting that nEOs take the main role in electric generation in the adult fish (Kirschbaum and Schwassmann, 2008). Although EO development and anatomy in the larval and adult stages have been extensively described, whether or not EO development initiates at embryonic stages has remained unclear.

The gymnotiform bluntnose knifefish, *Brachyhypopomus gauderio* (Giora and Malabarba, 2009; Giora et al., 2014), is one of the smallest knifefish species with a relatively short maturation time (about 4–6 months), making it a promising animal model for studying EO development. The species has already been extensively used for electrophysiological studies (e.g. Stoddard and Salazar, 2011; Matsushita et al., 2013) and for transcriptomics (Gallant et al., 2014; Guth et al., 2016). Previous studies have shown that the mEO in the *B. gauderio* develops at the ventral side of the tail myotome in the 6 days post-fertilization (dpf) larvae (Franchina, 1997). However, the embryonic origin of the EO primordial cells has not been characterized in any electric fish species to date. The aim of this study is to describe the embryonic and larval stages of development of *B. gauderio* and investigate the development of the EO during embryonic, larval, juvenile and adult stages of the species.

## 2 Material and methods

### 2.1 Fish husbandry and egg collection

*B. gauderio* were reared under laboratory conditions designed to mimic their natural habitat (26°C, pH7.3, reverse osmosis treated tap water reconstituted with salts to produce standardized synthetic fresh water, conductivity 300μS/cm, 16h light: 8h dark). One or two male and two females were kept in a 50 ​× ​50 ​× ​30cm tank filled with 60L water with circulation and aeration. Deionized water (8L) was added twice a week to change conductivity to enhance spawning. Adult fish were fed with frozen blood worms once a day. For egg collection, 15ml falcon tubes with 3mm holes were attached to the inside of the water tank. The fish occasionally spawn and deposit eggs in the tube through the holes during the night. All protocols used were permitted by the UK Home Office guidance to Animals Scientific Procedures Act.

### 2.2 Embryonic development

Collected embryos were cultured in 1.5L plastic tanks under a low flow-through condition of system water and with aeration. For imaging, live embryos were collected in a Petri dish with an agarose bed containing grooves filled with water. Images of embryos from fertilization until the early larval stage were captured using a stereo microscope Nikon SMZ1500. To capture detailed images of the older embryonic stages from 1 day post fertilization (dpf) to hatching, the chorion was enzymatically removed using pronase (Sigma-Aldrich) (1mg/ml in 0.5x Ringer for 10min). Embryos and larvae at 1-8dpf were anesthetized with MS-222 (tricaine methanesulfonate) solution (0.004%) and photographed.

### 2.3 Paraffin sectioning and histochemical staining

Whole embryos, larvae, and dissected adult tail tissues were fixed for 4–5 days in 4% paraformaldehyde (PFA) in PBS, subsequently dehydrated and embedded in paraffin wax (Sigma-Aldrich) using a Shandon Citadel Tissue Processor 2000 (Thermo Scientific), and sectioned to 5μm thickness using a rotary microtome (Leica Biosystems RM2125 RTS). Sections were stained with Haematoxylin and Eosin (H&E) using a Shandon Varistain 24-4 automatic slide stainer (Thermo Scientific), and embedded with Histomount (National Diagnostics). Images of histological sections were captured using a Zeiss Axioskop 40 microscope equipped with an Olympus color camera DP70.

### 2.4 Paraffin sectioning and immunohistochemistry

Paraffin sections were deparaffinized with Histo-Clear in two steps (5min each) rehydrated through an alcohol series (100%, 90%, 80%, 70% and 50%), and subsequently rinsed 5 times with tap water and washed with PBS containing 0.1% Triton X-100 (PBSTx) for 5min. The samples were incubated with blocking solution (5% BSA, 5% heat-inactivated Bovine Serum/HI-BS in PBS) for 20min then with the first antibody for 30min in a humid chamber. The mouse neuronal antibody, Zn-12 (Developmental Studies Hybridoma Bank) was used at a 1/20 dilution. The mouse Alpha tubulin antibody (Sigma-Aldrich T6199) was used at a 1/200 dilution. Slides were then washed three times with PBSTx for 5min each, and incubated with the secondary antibody, Biotinylated Anti-mouse IgG (H+L) (Vector Laboratories) for 30min. Slides were subsequently washed three times with PBSTx for 5min each before submerging in Stain Elite ABC reagent (Vector Laboratories) for 30min, washed three times with PBSTx for 5min each, followed by incubation with peroxidase substrate kit SK-4100 (Vector Laboratories) until the stain developed. To stop the staining, slides were rinsed with tap water and counterstained with Haematoxylin (1sec) and then with Eosin (1sec). Before mounting with Histomount, slides were dehydrated through an alcohol series (80% Industrial Methylated Spirits/IMS for 30sec, 90% IMS for 1min and 100% IMS for 2min) and with 100% ethanol for 2min followed by Histoclear for 5min. Slides were imaged using a Zeiss Axioskop 40 microscope.

### 2.5 Cryosectioning and fluorescent immunohistochemistry

For fluorescent immunohistochemistry, tissue samples of embryos and larvae were processed for cryosectioning. Fixed embryos and larvae were incubated in 30% sucrose in PBS overnight at 4°C. The samples were mounted in a mold with Neg-50TM frozen section medium (Fisher Scientific) and sectioned at 20–30μm using a cryostat (Leica CM 1950). Slides were washed twice with PBS for 10min each. The samples were incubated for 1h with blocking solution (5% BSA, 5% heat-inactivated Bovine Serum/HI-BS in PBS). Primary antibodies include anti-pan myosin MF20 (DSHB), Pax7 (DSHB) and pan-neural (anti HNK-1) Zn-12 antibodies at a 1/20 dilution respectively for overnight incubation. The slides were washed three times with blocking solution for 1h each and incubated overnight with Anti-mouse IgG Alexa 488 (1:1000) (Life Technology) and Hoechst (1:2000) (Life Technology). The slides were washed three times with blocking solution for 1h each and with PBSTx twice for 15min each. Mounting reagent (Invitrogen) was placed on tissues and covered with cover slip. Images were captured using a Zeiss LSM510 confocal microscope.

### 2.6 Plastic sectioning and MSS staining

For LR white resin embedment, embryos were fixed in 4% PFA, 0.1% glutaraldehyde in 0.1M PIPES (pH7.2), washed in the PIPES buffer and dH_2_O and dehydrated through an ethanol series (1 ​× ​50%, 70% and 2 ​× ​90% ethanol for 15min each) and then gradually embedded in LR white resin. The resin was cured at 50°C for 24h. For histological analysis the LR white-embedded embryos were sectioned at 1μm thickness. Sections were used for Multiple Stain Solution (MSS) staining (Polysciences) according to the manufacturer’s instruction.

### 2.7 Transmission electron microscopy (TEM)

Tails from embryonic and larval stages: 48 ​hours post fertilization (hpf), 60hpf, 72hpf 132hpf, 6.5dpf, 7.5dpf, 8.5dpf and 10.5dpf and normal adult: 7 months post fertilization (mpf) were processed for TEM analysis. All samples were fixed with 3% glutaraldehyde and 2% formaldehyde in 0.1M PIPES buffer (pH7.2) for 2h at room temperature then kept at 4°C until further use.

The samples were washed 3X for 5min each with 0.1M PIPES buffer, and then post-fixed for 1h in 1% OSO_4_ (reduced with 1.5% potassium ferrocyanide) in 0.1M sodium cacodylate (pH7.2). After 3 ​× ​5min washes with dH_2_O, samples were dehydrated through an ethanol series (1 ​× ​30%, 50%, 70%, 80%, 90%, 95% and 4 ​× ​100% ethanol, 10min each) and subsequently embedded in Spurr resin (TAAB Laboratories).

Cured resin blocks were trimmed with a razor blade then sectioned with a diamond knife using an ultra-microtome (Powertome Ultra cut, RMC). 70nm ultrathin sections were collected on pioloform-coated 100 mesh copper EM-grids (Agar Scientific) and contrasted with Reynold’s lead citrate (10min). The grids were imaged using a JEOL JEM 1400 TEM operated at 120kV and images captured with an ES 100W CCD digital camera (Gatan).

### 2.8 Live confocal imaging of the EO

For confocal imaging, live embryos and larvae were stained with 100μM BODIPY TR Methyl Ester (Invitrogen) in PBS for 30min, washed 3X in PBS for 5min each, and mounted in 1% low melting agarose with 0.02% MS222.

### 2.9 Amputation and regeneration of the caudal filament

Three females of *B. gauderio*, 106–113mm in total length were anesthetized with benzocaine (0.06 ​mg/L) and the caudal filament amputated with a sharp blade (Kirschbaum and Meunier, 1981, 1988; Unguez and Zakon, 1998). The samples were fixed in 4% PFA/PBS. After that the fish were moved to recovery tanks supplemented with Methylene Blue, and monitored until they recovered from anesthesia. Fish were kept overnight and observed for their recovery and health. The fish were then placed in a separated recirculation tank. After 2 weeks, the fish were anesthetized: the tail tip was cut again and sampled for analyzing regenerating tissues. These tissues were fixed, embedded in paraffin, sectioned and subject to HE-staining and immunohistochemistry as described before. All procedures used in this experiment followed the UK Home Office licensed protocols.

## 3 Results

### 3.1 Staging of the *B. gauderio* embryos and larvae

**Fig. 1:**
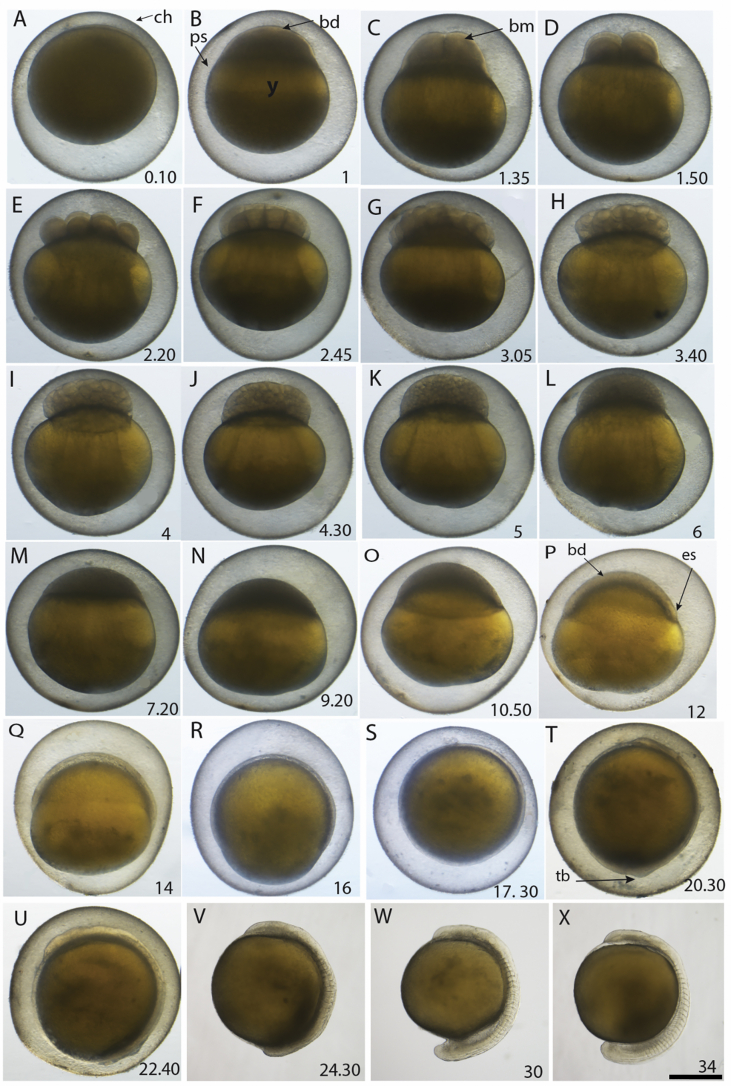
**Embryo development of *B. gauderio* - cleavage, blastula, gastrula and somitogenesis.** All images are lateral view with the animal pole up. A. Zygote 10min after fertilization. B. 1-cell stage (1h). C. 2-cell stage (1h35min). D. 4-cell stage (1h50min). E. 8-cell stage (2h20min). F. 16-cell stage (2h45min). G. 32-cell stage (3h05min). H. 64-cell stage (3h40min). I. 128-cell stage (4h). J. 256-cell stage (4h30min). K. 512-cell stage (5h). L. High stage (6h). M. Oblong stage (7h20 ​min). N. Sphere stage (9h20min). O. Dome stage (10h50min). P. 40% epiboly stage (12h). Q. 50% epiboly stage (14h). R. 75% epiboly stage (16h). S. 100% epiboly stage (17h30min). T. Bud stage (20h30min). U. 5 somite stage (22h40min). V. 10 somite stage (24h30min). W. 18 somite stage (30h). X. 24 somite stage (34h). Time is indicated as hours and minutes post fertilization at the bottom right side of each image. bd, blastodisc; bm, blastomeres; ch, chorion; ps, perivitelline space; y, yolk. es, embryonic shield; tb, tail bud. Scale bar ​= ​500μm.

**Fig. 2:**
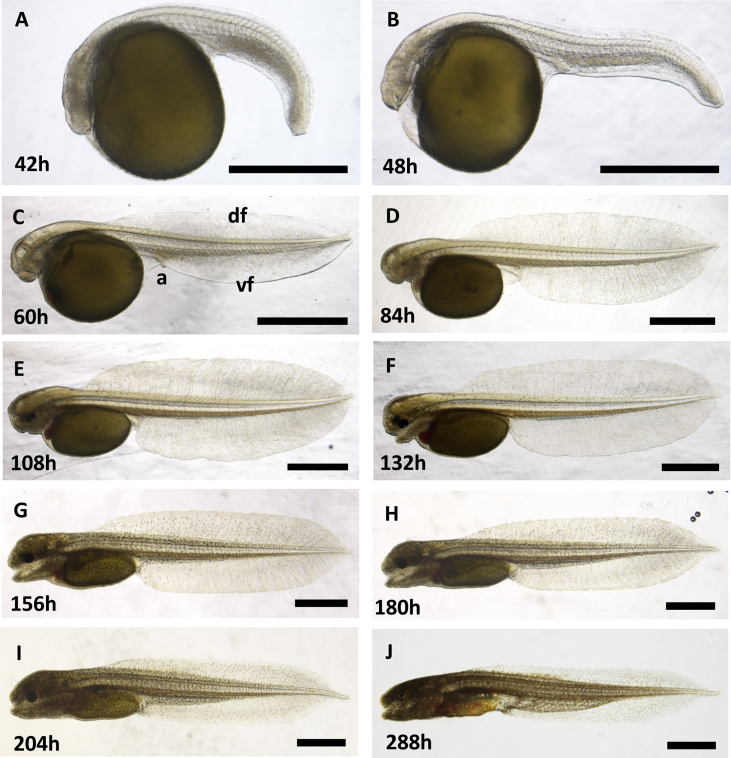
**Embryo development of *B. gauderio* – late somitogenesis and organogenesis stages**. All lateral view with anterior-left. Time is indicated as hours post fertilization: A 42h, B 48h, C 60h, D 84h, E 108h, F 132h, G 156h, H 180h, I 204h and J 288h. vf, vental fin; df, dorsal embryological fin fold; a, anus. Scale bars ​= ​1mm.

**Fig. 3:**
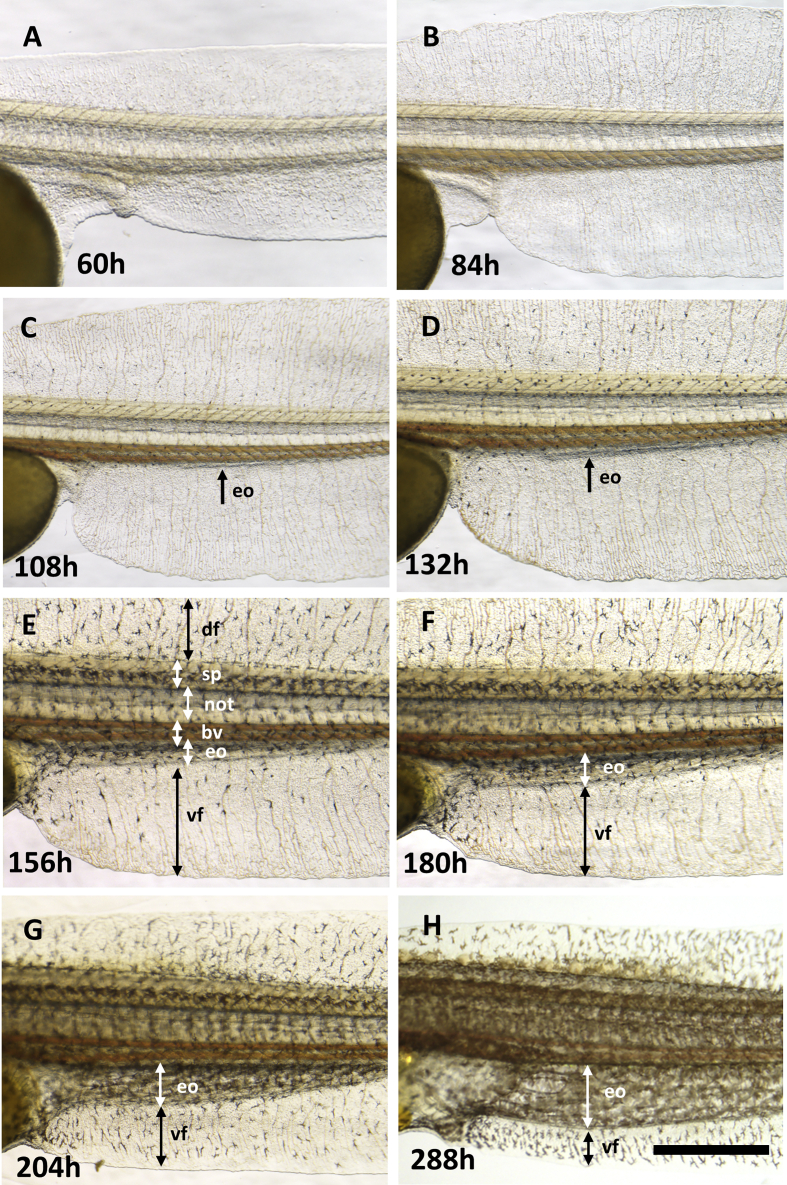
**Development of the mEO in the *B. gauderio***. All lateral view with anterior-left. Time is indicated as hours post fertilization: A 60h, B 84h, C 108h, D 132h, E 156h, F 180h, G 204h and H 288h. df, dorsal embryological fin fold; sp, spinal cord; not, notochord; bv, blood vessel; eo, electric organ; vf, ventral fin. Scale bars ​= ​1mm.

To use *B. gauderio* as a model animal for studying development of the EO, we first undertook a detailed characterization of its embryonic and larval stages (Fig. 1, Fig. 2, Fig. 3, Supp Fig. 1, Fig. 2, Supplementary text). The morphology of *B. gauderio* embryos at cleavage, blastula and gastrula stages is overall similar to that of cyprinid fish species such as zebrafish (Kimmel et al., 1995). However, after the somite stage, unique morphological characteristics become obvious in the developing fin. During this period, rapid growth of fins occurs on both the dorsal and ventral sides of the tail, with a broad fin structure developing by 60hpf (Figs. 2C and 3A). Soon after formation of the fin, the EO starts to develop at the border between the trunk and the ventral fin (Fig. 3C–H): the incipient EO is visible at the myotome-fin margin at 108hpf, and increases in size over the following days. Accordingly, the proportion of the EO area in the entire fin increases and the EO-free fin area decreases, suggesting that EO development occurs within the fin and the EO gradually expands toward the distal side of the ventral fin.

Besides the fins, the eye also shows a distinctive pattern of development: both retina and lens are smaller than in most other fish species (Fig. 2, Supp Fig. 2), consistent with the nocturnal lifestyle of *B. gauderio* relying mainly on electrolocation and electrocommunication. Embryos hatch at 4dpf and develop into swimming larvae (Fig. 2F–I, Supp Fig. 1D–G).

### 3.2 Dynamic translocation of EO primordia cells and rapid formation of layers of electrocytes during the late embryonic to early larval stage

**Fig. 4:**
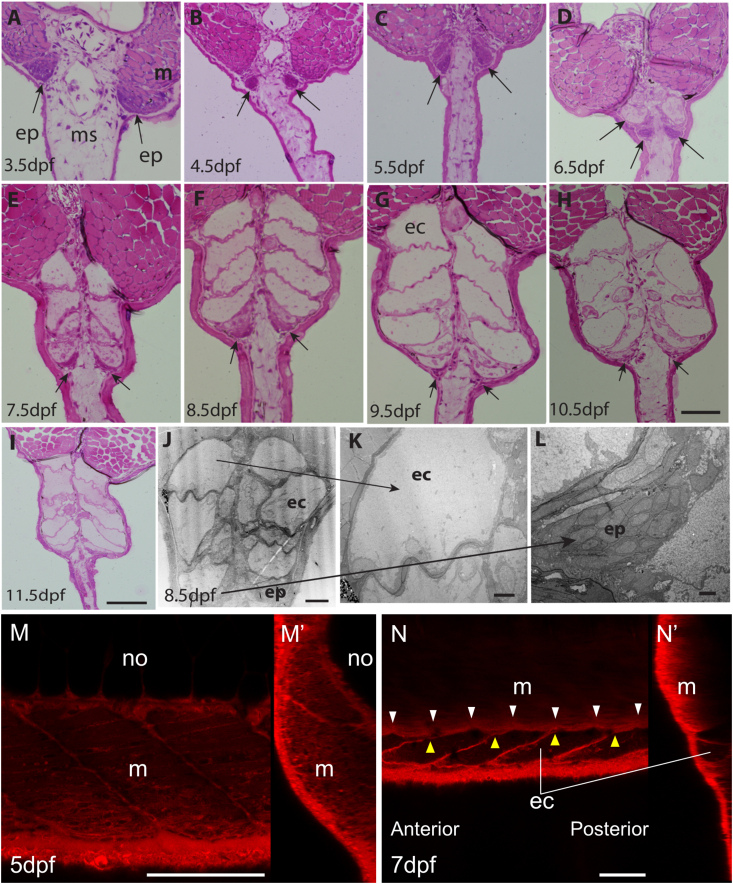
**EO** primordia **in the ventral somite translocate in the ventral fin and form the EO.** A-I, MSS staining of tails from a 3.5dpf embryo (A) and larvae at 4.5dpf (B), 5.5dpf (C), 6.5dpf (D), 7.5dpf (E), 8.5dpf (F), 9.5dpf (G), 10.5dpf (H) and 11.5dpf (I). Arrows shows that the EO primordia change location from embryonic stage (A, B) to larval stages (C–H) and become not recognizable at late larval stage (I). J-L, TEM images of 8.5dpf larva’s tail. M, N, Lateral confocal images of tail myotome and electrocytes in knifefish larvae at 5dpf (M) and 7dpf (N) stained with BODIPY TR Methyl Ester. M′, N′ Transverse sectional views. ep, dense cell mass of the EO primordia; ec, elctrocyte; m, myotome; ms, mesenchyme; no, notochord. White arrowheads, boundary between somites; yellow arrowheads, boundary between electrocytes. Scale bar ​= ​50μm in A-H; 100μm in I; 20μm in J; 5μm in K; 2μm in L; 100μm in M and N.

To elucidate the details of early stages of EO development, embryos and larvae were fixed every day from 3.5 to 11.5dpf, embedded in LR-white plastic resin and sectioned. These sections were used both for histological staining (MSS) and TEM (Fig. 4) allowing detailed observation of the developing EO. The MSS stained section reveals the formation of a pair of dense cell masses at the ventral end of the hypaxial muscle at 3.5dpf embryonic stage, which start to be separated from the muscle tissue at 4.5–5.5dpf (Fig. 4B and C). At 6.5dpf (Fig. 4D), when the dense cell mass is translocated more ventrally in the fin, the first electrocyte is formed between this mass and the hypaxial muscle. In the following days, this cell mass continuously translocates towards the distal tip of the fin, and each day an additional pair of electrocytes is formed at the position where the cell mass was located. The mass then gradually decreases in size. TEM images of larvae reveal a more detailed structure of the electrocytes (Fig. 4J-L), which can be identified as large multinucleated cells. (Fig. 4K). The densely packed cell mass is located at the leading edge of the developing EO and contains rounded cells with a relatively small amount of cytoplasm, typical of undifferentiated embryonic cells (Fig. 4L). These observations suggest that the described cell mass is the EO primordium that deposits cells to differentiate into electrocytes. To monitor real time development of the EO in vivo with 3D image, live embryos were labelled with BODIPY TR Methyl Ester and imaged by confocal microscopy (Fig. 4M and N). The images show that the EO is not detectable at 5.5dpf but is clearly visible at 7.5dpf. Individual electrocytes exhibit an elongated shape along the anterior-posterior axis. The border line between two electrocytes shows a posteriorly directed angled up-slope (Fig. 4N). This angled shape explains why the number of electrocytes differs in each transverse slice. The image also shows that boundaries between the somites and the boundaries between electrocytes do not align with each other. For instance, in Fig. 4N, there are six somites whereas there are only four electrocytes observed. This may suggest that the mechanism of EO development is not directly regulated by the process of somitogenesis.

### 3.3 The EO is derived from primordial cells in the somite

**Fig. 5:**
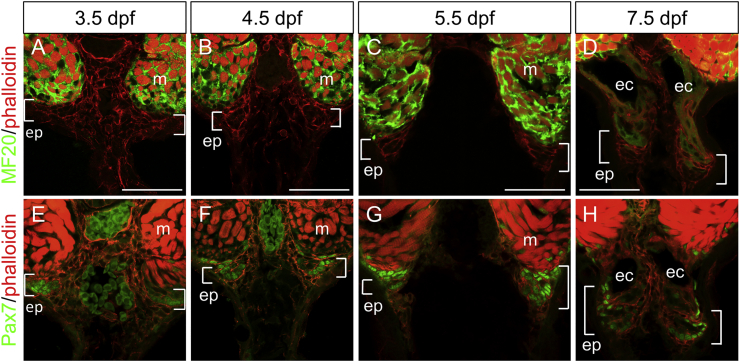
**EO primordium expressesPax7.** Immunohistochemistry of tail from 3.5dpf (A, E), 4.5dpf (B, F), 5.5dpf (C, G) and 7.5dpf (D, H) larvae. Sections were stained with MF20, anti-Myosin (A–D) and Pax7 antibodies (E–H) (green), and also counterstained with phalloidin (red). ep, EO primordium; ec, electrocyte; m, muscle. Scale bar ​= ​50μm.

To characterize and identify the origin of the EO primordia, embryos and early larvae were cryosectioned and stained with antibodies against a muscle myosin (MF20), and the transcription factor Pax7, a marker of muscle progenitor cells (Fig. 5). At the embryonic stage, 3.5dpf, most ventral myotomes express muscle myosin, whereas the outermost ventral end of the myotome has a pair of cell masses that shows reduced MF20 staining (Fig. 5A) and Pax7+ve nuclei (Fig. 5E). This domain is the equivalent to the region where the EO primordium was observed by TEM and MSS staining (Fig. 4). At 7.5dpf, the Pax7+ve EO primordium translocates towards the ventral fin (Fig. 5H). At that stage, the first electrocytes are observed between the ventral myotome and the primordium (Fig. 5D, H). These data suggest that the EO (mEO) is derived from the primordia that develop at the ventral somite prior to hatching.

### 3.4 Anatomy of the caudal filament of the adult fish

**Fig. 6:**
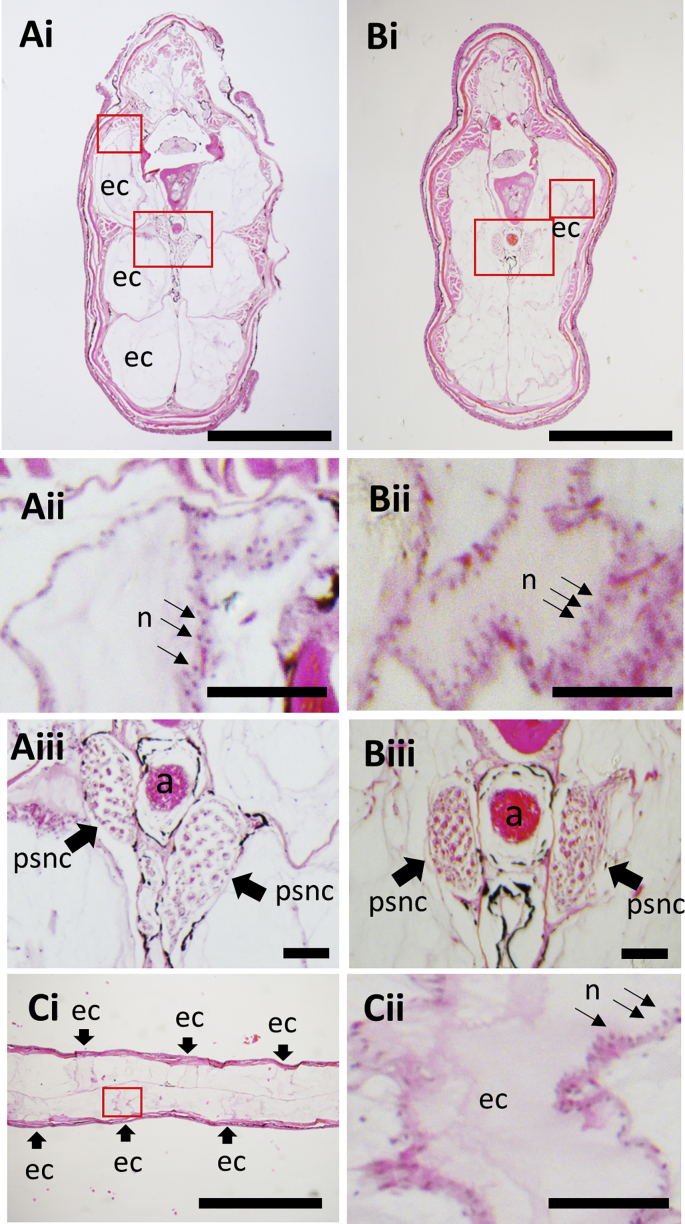
**Adult *B. gauderio* tail has mEO and a pair of pear-shaped nerve cords.** H&E staining of the middle (A) and posterior (B, C) parts of the caudal filament with transverse (A, B) and frontal sections (C). n, nuclei; psnc, pear-shaped nerve cord; a, aorta; ec, electrocyte. Scale bar ​= ​500μm in Ai, Bi, Ci; 50μm in Aii, Bii, Cii and 100μm in Aiii, Biii.

To elucidate the detailed structural organisation of the mature EO, the caudal filament in the posterior tails from adult fish was analyzed by H&E staining (Fig. 6). As previously reported in other species, the muscle is much smaller and the mEO occupies the largest area instead. The electrocyte in the EO in *B. gauderio* consists of extraordinarily large and highly multi-nucleated cells (Fig. 6 Aii, Bii). In the middle part of the caudal filament, the EO is separated into three sections (3 electrocytes) along the dorso-ventral axis (Fig. 6Ai) but in the distal end, there is no partition suggesting only one electrocyte is located in each section. When such distal caudal filament is sectioned with frontal view (Fig. 6C) a single large electrocyte is visible in the left or right side of the caudal filament. A pair of pear-shaped structures with a nerve-cord-like staining pattern was observed on either side of the dorsal aorta (Fig. 6Aiii, Biii). The location and morphology of the tissue are similar to that of the developing nEO in Apteronotids (Kirschbaum and Schwassmann, 2008)*.*

### 3.5 Multiple nuclei are periodically arranged at the periphery of the syncytial electrocyte

**Fig. 7:**
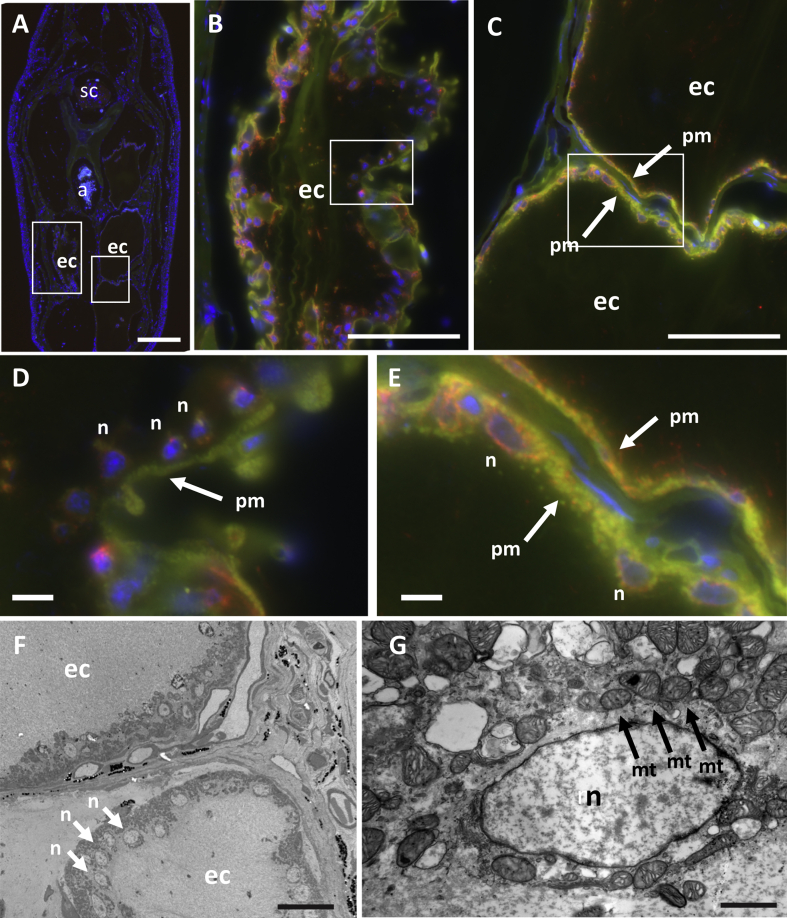
**Organelles are accumulated at the periphery of the plasma membrane in the EO.** Caudal filament of adult *B. gauderio* was analyzed with immunostaining with alpha-tubulin (red), phalloidin (green) and Hoechst nuclei staining (blue) (A–E), and with TEM (F, G). a, aorta; sc, spinal cord; ec, electrocyte. n, nucleus; mt, mitochondria; pm, plasma membrane. Scale bar ​= ​200μm in A; 100μm in B, C; 10μm in D, E; 20μm in F; 1μm in G.

To observe the structure of the mature mEO in more detail, the adult caudal filament was immunostained. Phalloidin staining reveals the actin filament localised at the inner plasma membrane, outlining the cell borders (Fig. 7A–C). With higher magnifications, images show that multiple cell nuclei are localised near the plasma membrane and aligned with relatively similar intervening distances (Fig. 7D and E). Microtubules are accumulated around these nuclei suggesting the microtubule network controls the distribution of nuclei around the cell membrane to separate them equidistantly, as seen in other syncytia, such as the yolk syncytial layer (Takesono et al., 2012). TEM images show a high density of mitochondria accumulated around the cell nuclei (Fig. 7G). In contrast to the peripheral accumulation of these organelles and cytoskeleton, the central part of the electrocyte is devoid of specific structures (Fig. 7C and F).

### 3.6 A pair of pear-shaped nerve cords is present in *B. gauderio* at a similar location to the nEO in Apteronotids

**Fig. 8:**
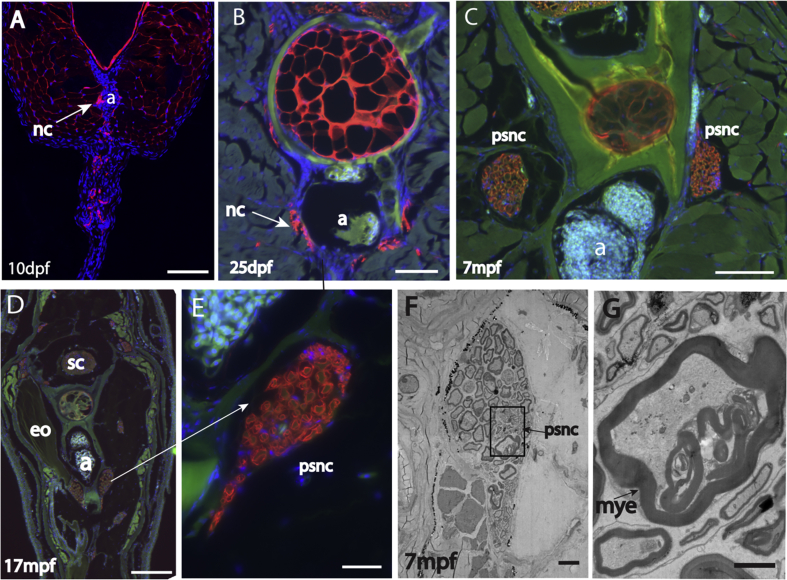
***B. gauderio* possess a pair of pear-shaped nerve cord** A-E. Immunohistochemistry of *B. gauderio* larvae and adult stained with neuronal antibody, Zn-12 (red). F.G. TEM images of the potential nEO showing intense myelination (mye) of the cells. a, aorta; ec, electrocyte; nc, nerve cord; psnc, pear-shaped nerve cord. sc, spinal cord; Scale bar ​= ​100μm in A, B; 500μm in D; 200μm in C; 50μm in E; 10μm in F; 2μm in G.

H&E staining reveals a pair of pear-shaped structures with nerve-cord-like morphology flanking the dorsal aorta in the caudal filament of the adult fish (Fig. 6Aiii, Biii). Staining of sections with a pan-neuronal antibody Zn-12, confirmed the neurogenic character of these pair-shaped structures (Fig. 8C–E). TEM images show the densely myelinated structure of neuronal cells (Fig. 8F and G). To elucidate the origin and developmental pattern of these neurogenic tissues, juvenile and larval tails were also sectioned and stained with Zn-12. The staining shows that even in 10dpf and 25dpf larvae, Zn-12-positive structures are visible at the side of aorta (Fig. 8A and B), suggesting primordia of the tissues develop at early larval stages. These data suggest that this neurogenic tissue is an equivalent structure to the Apteronotid neurogenic organ (Kirschbaum and Schwassmann, 2008).

### 3.7 The mEO and other major caudal filament tissues can regenerate following tail-amputation in adult *B. gauderio*

**Fig. 9:**
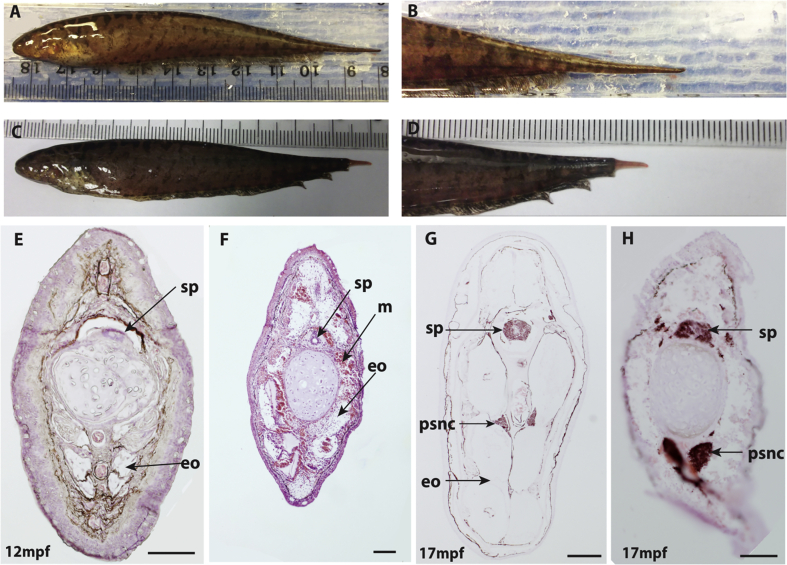
**mEO and other major tissues in the caudal filament can be regenerated after amputation.** Adult female fish before amputation (A, B, E, G) and two weeks after amputation (C, D, F, H). E, F. H&E staining of the caudal filament. G, H. Immunohistochemistry with neuronal antibody, Zn-12. eo, myogenic EO; sp, spinal cord; m, myotome; psnc, pear-shaped nerve cord. Scale bar ​= ​200μm in E, F, G; 100μm in H.

Regeneration of adult mEOs following tail-amputation of knifefish species has previously been described (Kirschbaum and Meunier, 1981, 1988; Unguez and Zakon, 1998). This system provides a powerful experimental model in which to analyse the molecular and cellular mechanisms of EO development. We tested if mEOs can regenerate in the *B. gauderio* by cutting the caudal filament (Fig. 9A and B). Two weeks after amputation, 7mm of newly formed caudal filament was regenerated (Fig. 9C and D). H&E staining of the newly regenerated caudal filament confirmed that mEOs, muscle and cartilage can regenerate. Zn-12 labelling reveals that the pear-shaped nerve cords and spinal cord can also regenerate. These data suggest that adult *B. gauderio* can develop major tissues in the caudal filament including, mEOs and nerve cords.

## 4 Discussion

### 4.1 Embryonic and larval development of the *B. gauderio*

Embryonic development of *B. gauderio* shows many similarities to that of zebrafish including the proportions of the blastoderm/yolk, morphology of blastomeres, egg color and relatively soft & thin chorion, which is easily degraded by pronase (Fig. 1). This is consistent with South American knifefish (Gymnotidae) and zebrafish (Cyprindae) belonging to relatively closely related families compared to many other teleost species (e.g. elephant nose fish, medaka, killifish, salmonidae). At late somite stage, however, differences become more apparent: *B. gauderio* develops a wide ventral fin in the tail, which becomes the platform of EO development.

### 4.2 The EO primordium arises at the ventral edge of the ventral myotome at late embryonic stage, migrates into the ventral fin and develops into the EO at early larval stages

We identified the EO primordia arising at embryonic stages before hatching. Detailed observation of sections from embryonic to larval stages revealed translocation of the EO primordia from the ventral myotome into the ventral fin to form the EO. A pair of newly formed electrocytes appears daily between 6.5 and 9.5dpf and forms approximately 6 layers of electrocytes. EO primordium cells were previously described in the electric eel (Schwassmann et al., 2014) in which a dense cell mass called electromatrix, similar to the *B. gauderio* EO primordium, was identified in the 100 somite stage larva. The layer pattern of the newly formed EO at early larval stages was also similar. It therefore seems likely that the mechanisms underlying mEO development is conserved between *B. gauderio*, electric eel and possibly in other Hypopomidae fish species. Previous studies of regeneration of the EO in adult knifefish, *S. macrurus*, suggested that Pax7+ve muscle stem cells are localised around the EO and can differentiate into the mEO during tail regeneration (Weber et al., 2012). Our immunohistochemistry data revealed that the Pax7+ve cell mass at the edge of the myotome migrates into the ventral fin and develops into the EO. This suggests that similar mechanisms are conserved between development of the EO during the embryo to larva transition and during regeneration of the EO in the adult fish. However, the mechanism by which only the ventral most myotomal cells separate their fate from muscle and differentiate into the EO remains unclear.

### 4.3 *B. gauderio* possess pear-shaped nerve cord

We found a pair of pear-shaped nerve cords (PSNC) flanking the dorsal aorta in the tail. The morphology and position of these structures are similar to the nEO in the Apteronotids. This suggests that the PSNC has a common evolutionary origin to the nEO and might have overlapping function, related to regulating electric discharge. Although the morphology of the *B. gauderio* PSNC is similar to the Apteronotid nEO, it is more similar to the larval nEO than the adult nEO. The adult nEO reported in *Apyrtonotus leptorhyncus* is a very large structure that fills up a major part of the caudal filament (Kirschbaum and Schwassmann, 2008) whereas PSNC in *B. gauderio* occupies a small region in the caudal filament and rather similar to the developing nEO in the larval stage of the *Apteronotus leptorhyncus*. It is therefore possible that the PSNC in *B. gauderio* may not function as an EO, but rather, be the axons of motoneurons that innervate the mEO. Further anatomical, genetic and functional neurophysiology analyses of the PSNC in the *B. gauderio* and nEO in Apteronotids will be required to shed further light on the evolution and function of these tissues.

### 4.4 Regeneration of the mEO and other tail tissues in the adult fish

As mentioned above, the Gymnotiform knifefish is an excellent model for the analysis of the molecular and cellular basis of tail regeneration as it can regenerate all or most tissues including the EO, muscle, spinal cord, spine and blood vessel (Kirschbaum and Meunier, 1981, 1988; Unguez and Zakon, 1998; Weber et al., 2012). In this work, we have shown that besides these tail tissues, the pear-shaped nerve cords at either side of the dorsal aorta can also regenerate. Therefore, adult tail represents an alternative and powerful tool to uncover the molecular basis of the development of the EO and associated nervous systems in this species. For instance, it is possible to isolate larger numbers of concentrated EO primordia cells from the adult regenerating tissue compared to the embryo/larvae, facilitating preparation of samples for transcriptome analyses by RNA-seq. Such analyses of adult mEO from several electric fish species have already been reported (Gallant et al., 2014). More recently, Guth et al. (2016) reported transcriptome analysis of regenerating tail showing stage specific gene expression during the developmental process of regenerating mEO. It would be interesting to compare such transcriptomes of the EO primordia and developed EO both in the mEO to identify and characterize key genes that are transiently expressed in the primordia and regulate the development of these tissues.
