# Supplementary material for: Development of the electric organ in embryos and larvae of the knifefish, Brachyhypopomus gauderio
Source: Dev Biol. 2020 Oct 1;466(1-2):99–108. doi: 10.1016/j.ydbio.2020.06.010 (PMC7507958; doi:10.1016/j.ydbio.2020.06.010)

**Supplementary text**

Developmental stages of *B. gauderio*:

Formation of the blastoderm (0-1 hpf): The fertilised egg shows sphere shape with shady yellowish yolk in which animal-vegetal polarity is not clear yet (A). However by 1 hpf, the blasdoderm is formed showing a transparent and clearly separated cell, blastomere, at the animal pole and becomes distinguishable from the rest of the yolk.

Cleavage stages (1-5 hpf): During the first 5 hours, cleavage occurs in the blastoderm to multiply blastomeres (Fig. 1A-K).

Blastula stage (Fig. 1L-O): At blastula stage, shape of the blastoderm changes:

High stage (6 hpf): The blastoderm is slightly elongated along the Animal-Vegetal pole (Fig. 1L).

Oblong (7.20 hpf): The blastoderm becomes slightly flatter (Fig. 1M).

Sphere (9.20 hpf): The interface between the blastoderm and yolk changes from flat to slightly round surface (Fig. 1N).

Dome stage (10.50 hpf): Roundness of the interface between the blastoderm and yolk is more enhanced (Fig. 1O).

Gastrula stage (Fig. 1P-T): At gastrula stage, the blastoderm expands toward the vegetal pole and cover the yolk:

Shield stage (12 hpf): The blastoderm covers approximately 45% of the yolk. The embryonic shield is visible at the dorsal blastoderm margin (Fig. 1P).

60% epiboly stage (14 hpf): The blastoderm covers 60% of the yolk (Fig. 1Q).

80% epiboly stage (16 hpf): The blastoderm covers 80% of the yolk (Fig. 1R).

100% epiboly stage (17.30 hpf): The blastoderm covers 100% of the yolk (Fig. 1S).

Bud stage (20.30 hpf): small head and tail bud are formed at the Animal and Vegetal poles respectively (Fig. 1T).

Somite stages (22-48 hpf) (Fig. 1U-X, Fig. 2A,B): During the somitogenesis stages, number of the somite increases. Head size gradually increase and the body (head-trunk-tail) elongate along the anterior-posterior axis.

Fin stages (60-204 hpf) (Fig. 2C-I, Fig.3A-G, Supp Fig. 1, Supp Fig. 2): Typical morphology of the dorsal and ventral fin develops at these stages. Head size and body length further increase. Pigmentation in the eyes is observed at 108 hpf (Fig. 2E, Supp Fig. 2C). Hatching occurs around the 84 to 108 hpf (Fig. 2D-E). EO develops at the border between the tail and ventral fin (Fig. 3). The shape of the yolk is initially spherical (Fig. 2B) but gradually become thinner oval shape (Fig. 2C-H). The jaw gradually develops from 84 to 204 hpf (Supp Fig. 2).

12dpf larvae (288 hpf) (FIg. 2J): Yolk is reduced and becomes flatter. Jaw is further developed and consequently the face becomes thinner and longer. EO is expanded and occupies more than 50% of the ventral fin area (Fig. 3H).

**Supplementary Figure Legends**

**Supplementary Figure 1.**

**Embryo development of *B. gauderio* - somitogenesis and organogenesis stages***.* **Dorsal view.** Time is indicated as hours post fertilisation: (A) 60 h, (B) 84 h, (C) 108 h, (D) 132 h, (E) 156 h, (F) 180 h, (G) 204 h and (H) 288 h. Scale bars = 1 mm.

**Supplementary Figure 2. Head development in the *B. gauderio****.* All lateral view with anterior-left. Time is indicated as hours post fertilisation: (A) 60 h, (B) 84 h, (C) 108 h, (D) 132 h, (E) 156 h, (F) 180 h, (G) 204 h and (H) 288h. mhb, mid-hindbrain boundary; ov, otic vesicle; h, heart. Scale bars = 1 mm


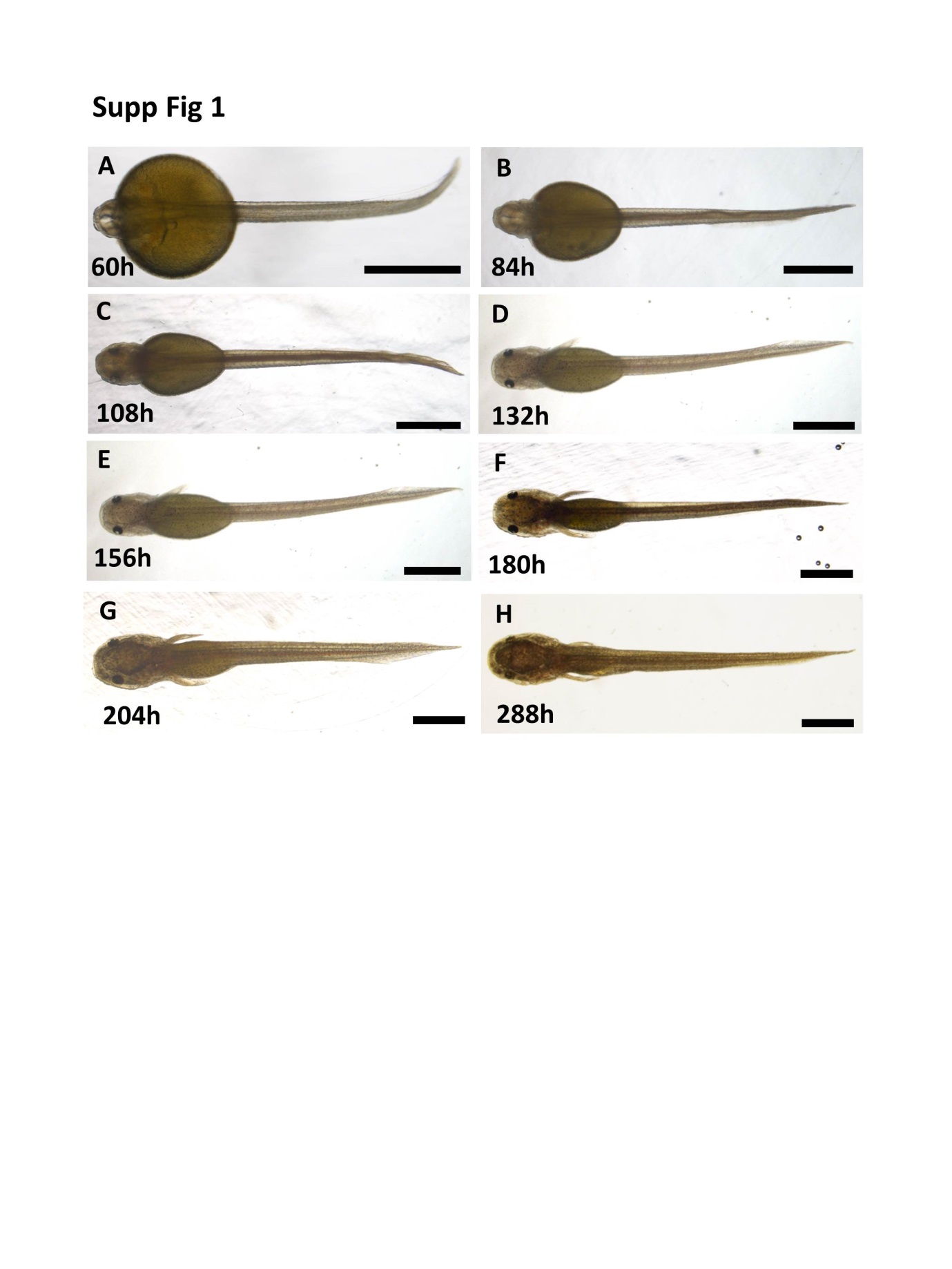


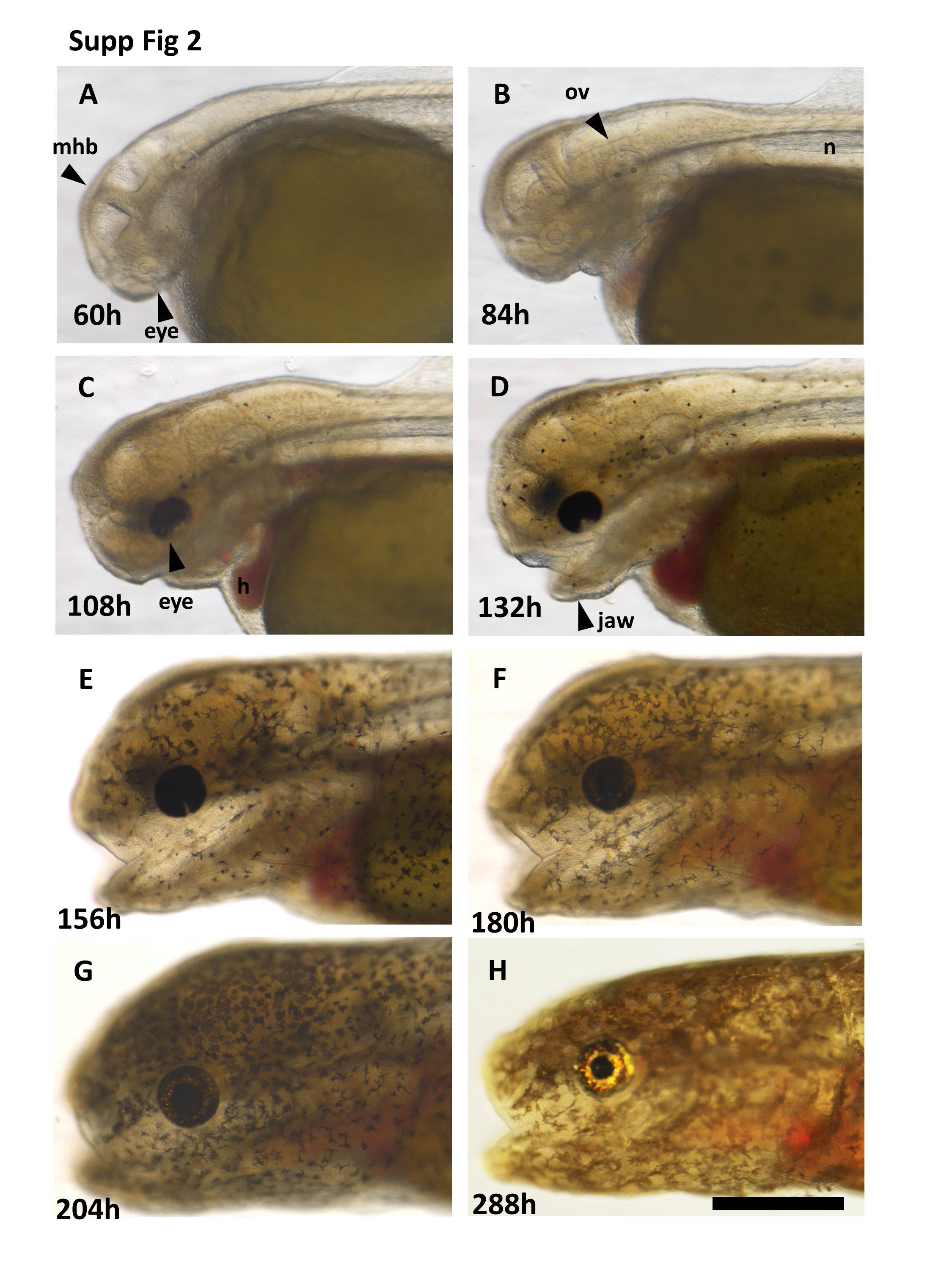

Supplement: Multimedia component 1 [file mmc1.docx]
